# Episodic herbivory, plant density dependence, and stimulation of aboveground plant production

**DOI:** 10.1002/ece3.6274

**Published:** 2020-04-24

**Authors:** Mark E. Ritchie, Jacob F. Penner

**Affiliations:** ^1^ Department of Biology Syracuse University Syracuse NY USA

**Keywords:** aboveground primary production, density dependence, episodic herbivory, grasslands, grazing, herbivory, herbivory event, plants, relative growth rate, Serengeti

## Abstract

Herbivory is a major energy transfer within ecosystems; an open question is under what circumstances it can stimulate aboveground seasonal primary production. Despite multiple field demonstrations, past theory considered herbivory as a continuous process and found stimulation of seasonal production to be unlikely. Here, we report a new theoretical model that explores the consequences of discrete herbivory events, or episodes, separated in time. We discovered that negative density (biomass) dependence of plant growth, such as might be expected from resource limitation of plant growth, favors stimulation of seasonal production by infrequent herbivory events under a wide range of herbivory intensities and maximum plant relative growth rates. Results converge to those of previous models under repeated, short‐interval herbivory, which generally reduces seasonal production. Model parameters were estimated with new and previous data from the Serengeti ecosystem. Patterns of observed frequent and large magnitude stimulated production in these data agreed generally with those predicted by the episodic herbivory model. The model thus may provide a new framework for evaluating the sustainability and impact of herbivory.

## Graphical abstract

The issue of whether herbivores can stimulate plant production remains controversial. Here we develop a theoretical framework for understanding how discrete or episodic herbivory events can stimulate plant production under a wide variety of conditions without increasing nutrient availability. The model predictions, based on new data for relative growth rates of grassland plants, agree reasonably well with previously published data for grazing impacts on above‐ground production in the Serengeti.

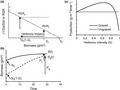

## 1 INTRODUCTION

As the first step in the transfer of energy from primary producers to consumers in ecosystems, herbivory is a key and ubiquitous trophic interaction (Milchunas & Lauenroth, 1993). An important, often‐studied, but poorly understood phenomenon is the effect of herbivores on aboveground net primary production (ANPP). While “productivity” is usually assumed to be an instantaneous rate (gross C assimilation minus C losses from plant tissue loss plus respiration), in habitats where plant biomass accumulates from instantaneous productivity integrated over time, ANPP is measured as an accumulation of biomass divided by the time required to achieve that accumulation. Such treatment is typical for grasslands (Frank, Kuns, & Guido, 2002; Frank, Wallen, & White, 2016; McNaughton, Milchunas, & Frank, 1996), forests (Berryman, Stenseth, & Isaev, 1987; Pastor & Naiman, 1992), and other habitats such as those dominated by aquatic macrophytes or seagrass beds (van Tussenbroek & Morales, 2017). Here, we focus on the effects of herbivores on ANPP as measured by what we call seasonal production, or the accumulation of biomass over a period of growth, or season, divided by the length of the growth period.

While many studies have shown that herbivores decrease seasonal production (Milchunas & Lauenroth, 1993), other studies have shown that moderate herbivory intensities can stimulate seasonal production (Detling & French, 1979; Frank, Depriest, McLauchlan, & Risch, 2011; Hilbert, Swift, Detling, & Dyer, 1981; Lebon, Mailleret, Dumont, & Grognard, 2014; Luo et al., 2012; de Mazancourt, Loreau, & Abbadie, 1998; Williamson, Detling, Dodd, & Dyer, 1989; Yamauchi & Yamamura, 2004). What is less well known is under what circumstances herbivory can stimulate aboveground production (Noy‐Meir, 1993). This question has been poorly addressed for nearly 50 years (Arsenault & Owen‐Smith, 2002; Belsky, Carson, Jensen, & Fox, 1993; de Mazancourt et al., 1998; McNaughton, 1976) and is still unresolved (Briske, 1993; DeAngelis & Huston, 1993; Zegler, Brink, Renz, Ruark, & Casler, 2018). Herbivory technically will stimulate seasonal production (overproduction) above that in the absence of herbivory when it elevates both plant relative growth rate and total biomass accumulation rate. Herbivory may increase plant relative growth rate by reducing biomass and intraspecific competition for limiting resources, such as light (Alward & Joern, 1993; Hilbert et al., 1981; Huisman et al., 1999; Noy‐Meir, 1975; Schwinning & Parsons, 1999). Alternatively, herbivory may stimulate productivity by increasing nutrient recycling (de Mazancourt et al., 1998; McNaughton, Banyikwa, & McNaughton, 1997; Ritchie, Tilman, & Knops, 1998). Both mechanisms imply a potential interaction between herbivory, resource limitation and intraspecific competition within plants, (de Mazancourt et al., 1998; van Staalduinen, Dobarro, & Peco, 2010; Wise & Abrahamson, 2005). However, theoretical studies, which have largely treated plant growth and herbivory as a continuous process, generally have found herbivore stimulation of productivity unlikely in the absence of herbivore‐enhanced nutrient cycling (DeAngelis, 1992; Loreau, 2010; de Mazancourt et al., 1998).

Here, we consider herbivory to be episodic that is an event in which some fraction of biomass is removed (herbivory intensity), and production is the biomass accumulation (growth) over some time interval both before and following that event. We argue that herbivory can be inherently episodic, particularly in terrestrial environments where herbivores consume a significant fraction of production. Individual plants may not experience daily or even weekly loss of tissue; rather, plants often lose a significant fraction of their biomass over short time periods. For example, plants lose tissue as bites, sometimes several in succession (Belovsky, 1986; Hobbs, Gross, Shipley, Spalinger, & Wunder, 2003) to mammalian herbivores, and specialist insect herbivores may impose most of the consumption of their host plant in their final instar of development (Agrawal, 1998; Brown, Gange, Evans, & Storr, 1987). Episodes of herbivory can occur for both herbaceous and woody plants in terrestrial environments (Broadbent, Bork, Cooke, & Willms, 2019; Broadbent, Bork, & Willms, 2018; Call & St Clair, 2018; Herrero‐Jauregui, Schmitz, & Pineda, 2016; Huttunen et al., 2013; Mudongo, Fynn, & Bonyongo, 2016; Teague, Dowhower, & Baker, 2016) as well as plants in aquatic systems (van Tussenbroek & Morales, 2017). Fisheries models suggest that pulsed harvests cannot stimulate yields (Braverman & Mamdani, 2008). However, this model includes no explicit feedback between harvest and population growth rate, as might occur where plant growth is resource‐limited and herbivory affects access to resources. Such feedback appears to be important but no theoretical framework for episodic herbivory exists from which to formulate and test hypotheses about episodic herbivory effects on plants and the ensuing plant responses.

To explore the consequences of episodic herbivory, we modify a previous model of plant response to herbivory (Hilbert et al., 1981), based on an assumption of exponential plant growth, to consider effects of herbivory on density (biomass)‐dependent growth by plants. We then connect this more general approach to explicit resource limitation of plants to explore how resource supply and availability might influence plant density dependence and the impacts of herbivores. We derive first a simple description of plant growth as resource‐limited and therefore density‐dependent, and then explore the conditions of herbivory intensity, frequency, plant maximum relative growth rate, biomass at the time of herbivory, and steady‐state plant biomass in the absence of herbivory that influence whether herbivores stimulate plant production by herbivores.

We confront the episodic herbivory model's predictions with both new and published field data from the Serengeti ecosystem (McNaughton, 1985; Ritchie, 2014). Measurements of community (all species’) biomass at monthly intervals in fenced plots were used to estimate relationships between relative growth rate, RGR, and biomass and to estimate maximum RGR across seven sites that differ in rainfall and soil nutrients. These data were used to parameterize the model of episodic herbivory, which was then tested with reported herbivory (grazing) intensity and grazed and ungrazed production at twenty sites across the Serengeti from the classic study by McNaughton (McNaughton, 1985).

## 2 METHODS

### 2.1 Models

#### 2.1.1 Herbivory and biomass dynamics

We begin with a classic logistic description of the density‐dependent rate of change in plant biomass

(1) $$\text{dS}/\text{dt}=S\;r\left(1-S/S_{K}\right)$$

where *S* is biomass (mass/area), *r* is a maximum relative growth rate under ideal conditions (mass^.^mass^−1.^time^−1^), *t* is time, and *S_K_* is the steady‐state biomass in the absence of herbivory. Mass‐specific, or relative, growth rate, *RGR* is given as

(2) $$RGR=\left(1/S\right)\left(dS/dt\right)\;=r\left(1-S/S_{K}\right)$$

**Figure 1:**
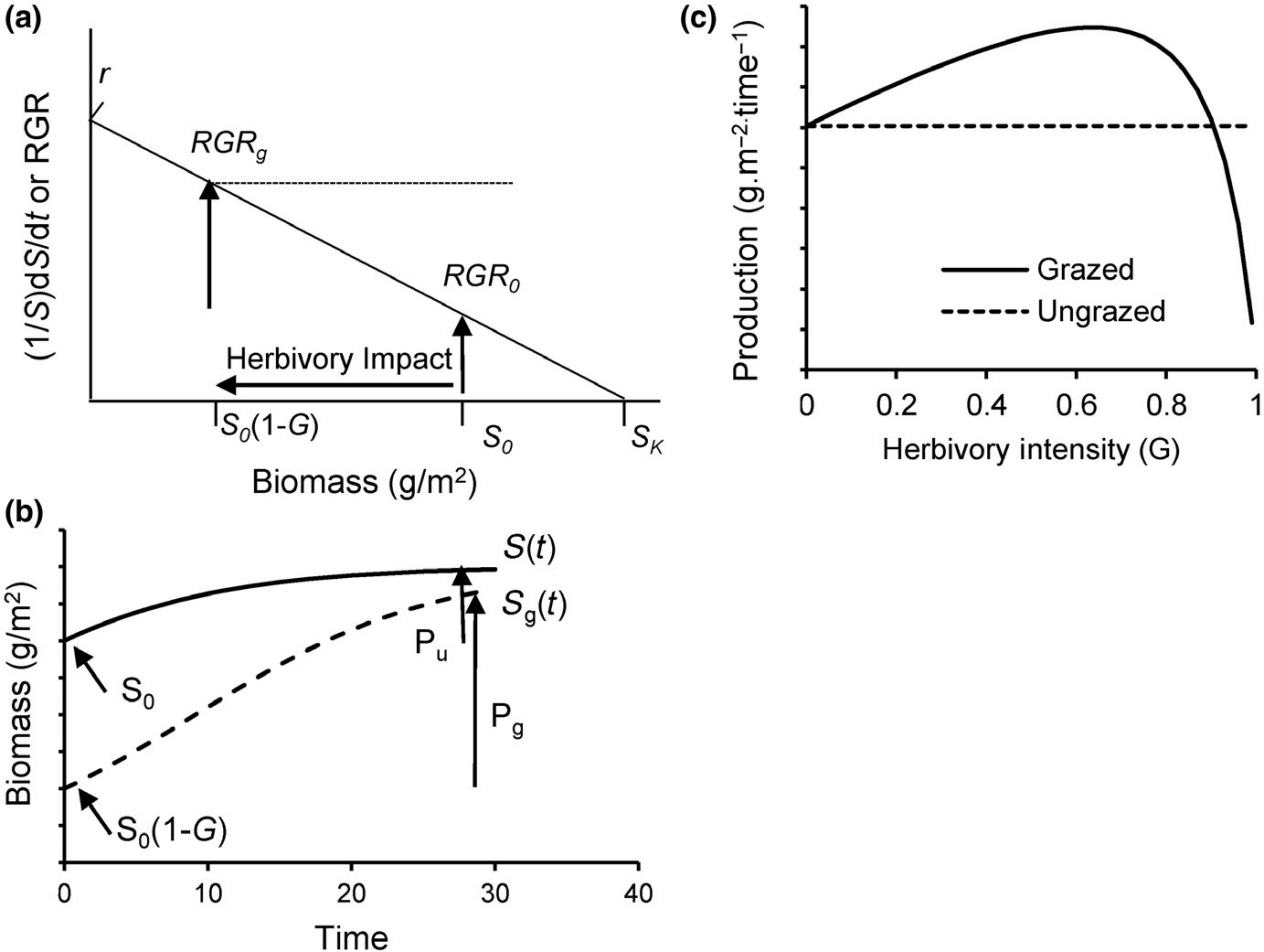
Graphical representation of the theoretical episodic herbivory model. (a) Under density‐dependent plant dynamics, relative growth rate *RGR* (solid line) declines from a maximum ate *r* at *S* = 1, to zero at steady‐state biomass without herbivory, *S_K_*. Biomass removal as a proportion *G* (herbivory intensity) by herbivores from an initial biomass *S*_0_ at the time of the herbivory event to yield a biomass immediately following herbivory *S*_0 _(1‐*G*). The expected increase in *RGR* from that at initial biomass *S*_0_ (*RGR*_0_) to that at biomass following herbivory *S*_0_(1‐*G*), *RGR_g_*, reflects the reduction in density‐dependent effect of biomass on *RGR*. (b) Biomass accumulation over a time *t* without herbivory (solid curve) and following an herbivory event that removes a proportion *G* of biomass (intensity) (dashed curve). Seasonal production is estimated from the difference between biomass at time *t* without herbivory [*P_U_* = *S*(*t*) – *S*_0_] and following herbivory [*P_g_* = *S_g _*(*t*) – *S*_0 _(1‐*G*)]. (c) Calculation of the difference in seasonal production over time *t* between plants following the herbivory event (dashed curve) and plants without herbivory (solid horizontal line)

Note that all these parameters are measurable in the field and have ecological meaning, including *r*, which represents a plant trait. The value of *r* can be attributed to a single species in the case of a specialist herbivore on its host plant or to an agricultural monoculture grazed by livestock, or it can reflect a community‐weighted mean trait from a multi‐species assemblage (Muscarella & Uriarte, 2016). *RGR* is assumed to decline linearly with increasing biomass, presumably due to greater resource limitation at higher biomass (Figure 1a). The linear assumption is an approximation that allows time‐dependent trajectories of biomass to be solved analytically and thus generally. Field results reported here support this approximation.

### 2.2 Episodic herbivory

From some initial biomass *S*_0_ at the time of herbivory, biomass *S*(*t*) at a future time *t* can be found by solving the separable differential equation in Equation (1), yielding

(3) $$S\left(t\right)=\frac{S_{K}S_{0}}{S_{K}e^{-rt}+S_{0}\left[1-e^{-rt}\right]}$$

An herbivory event removes a proportion *G* (herbivory intensity) of biomass at initial value *S*_0_, yielding biomass following herbivory of *S*_0 _(1‐*G*) (Figure 1a). Thus, we can determine biomass at a future time *t* following the herbivory event as

(4) $$S_{g}\left(t\right)=\frac{S_{K}S_{0}\left(1-G\right)}{S_{K}e^{-rt}+S_{0}\left(1-G\right)\left[1-e^{-rt}\right]}$$

Inspection of Equation 4 reveals that increasing herbivory intensity, *G*, has both positive and negative effects on the factors affecting productivity. A positive effect is that herbivory increases *RGR* (Equation 2) by reducing biomass and thus the strength of biomass density dependence. A negative effect arises from the reduction in available biomass for generating new tissue, which reduces the absolute biomass growth rate (Figure 1b).

Growth (increase in biomass) of plants from the different initial biomasses in the absence of herbivory gives production, measured over a time interval *t*:

(5) $$P_{u}=\;\left(S\left(t\right)\;-S_{0}\right)/t$$

Note that production is low if biomass is near *S_K_* due to the balance of growth with tissue loss due to resource limitation. This is commonly observed in forests where biomass remains approximately constant over the season following initial leaf‐out in the absence of herbivory (Xiao et al., 2004). Production under herbivory, *P_g_*, is therefore

(6) $$P_{g}=\;\left[S_{g}\left(t\right)\;-S_{0}\left(1-G\right)\right]/t$$

Production under herbivory (Equation 6) is a nonlinear function of herbivory intensity *G* (Figure 1c). Light herbivory intensity (small *G*) does not reduce biomass enough to achieve significant release from density‐dependent biomass inhibition and increase *RGR*. If *r* is high enough, and *t* long enough, intermediate *G* can lead to higher productivity than in the absence of herbivory. However, under intense herbivory (large *G*) the reduction in biomass can be large enough that the absolute increase in biomass is reduced, despite a higher RGR, resulting in herbivore‐induced decline in production.

**Figure 2:**
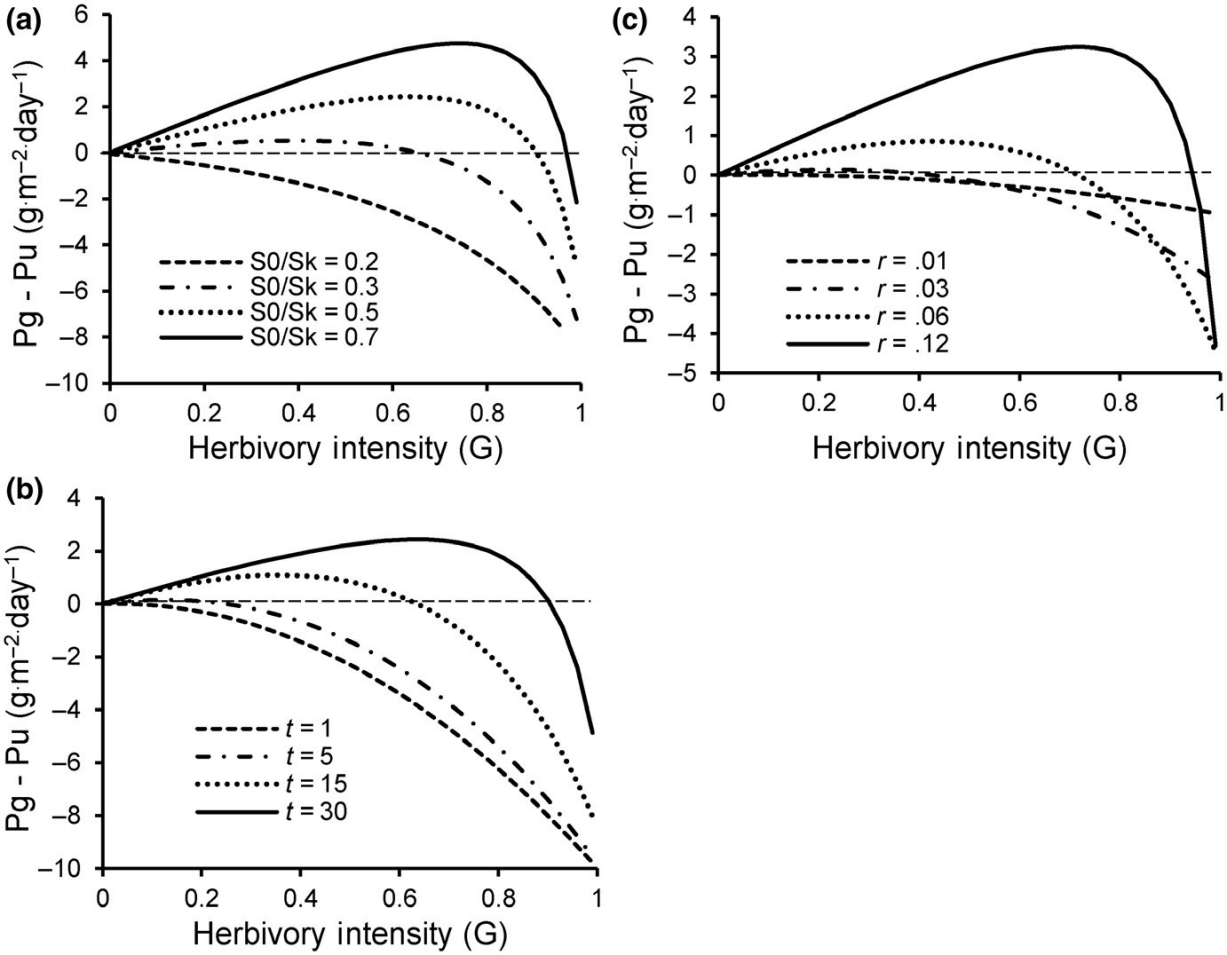
Herbivore effects on production as a function of herbivory intensity, *G*, as driven by (a) initial relative biomass *S*_0_/*S_K_*, (b) time interval following herbivory *t*, and (c) maximum relative growth *rate* or *r*. The horizontal dashed line represents equal productivity

Stimulation of production (overproduction) occurs when Δ*P* = *P_g_* – *P_u_* > 0 (Figure 1a,b). Substituting Equations 3 and 4 for the time‐dependent biomass functions in (5) and (6) yields.

(7) $$\Delta P=S_{0}\left\{\left(1-G\right)\left[\left(\frac{1}{Q-fG}\right)-1\right]-\left[\frac{1}{Q}-1\right]\right\}/t$$

where *Q* = e^−^ *^rt^* + *f* and *f* = (*S*_0_/*S_K_*)(1‐e^−^ *^rt^*). The dependence of Δ*P* on herbivory intensity is modified by three factors (Figure 2): how close initial biomass at the time of herbivory is to steady‐state biomass in the absence of herbivory (*S*_0_/*S_K_*) (Figure 2a), time *t* following the herbivory event (Figure 2b), and the maximum *RGR*, *r* (Figure 2c).

### 2.3 Field Measurements

As an example, empirical data measurements to estimate model parameters, such as *r*, *t*, *G*, and *S*_0_/*S_K_*, were obtained from two different studies in the Serengeti ecosystem, including Serengeti National Park in Tanzania (2^o^16’S, 34^o^56’E) and Masai Mara National Reserve in Kenya (1^o^29’S, 35^o^5’E).

### 2.4 Community biomass relative growth rate

Relative growth rate of community biomass, or that of all species, as a function of biomass was determined from biomass measurements made once, at least 27 days apart, in each of consecutive months April, May, and June in 2000, 2001, and 2002 in three 4 × 4 m fenced plots at each of seven sites from the long‐term grazing exclosure (LTGE) experiment (Ritchie, 2014; Veldhuis et al., 2019). Four 25 × 25 cm quadrats were clipped to the ground surface in each 4 × 4 m plot, with green material separated from litter. Different quadrats were clipped from different locations within plots in each month. Green material was air‐dried at 45°C for a week and then weighed. Sites varied in mean annual rainfall from 490 to 890 mm/yr, soil N from 0.05% to 0.22% and soil P from 0.005% to 0.15% (Ritchie, 2014).

*RGR* is operationally defined as ln (*S_m_*/*S_m‐1_*)/*t* where *S* is biomass, *m* is month, and *t* is the number of days between biomass samples. Maximum community biomass relative growth rate, *r*, was estimated as the intercept of a regression of *RGR* versus* S_m‐1_* calculated for all twelve paired monthly samples (April to May, May to June, three plots, and two years). Points are not independent because the independent variable *S_m‐1_* is also present in the calculation of the dependent variable *RGR*. However, the regressions, conducted separately for each of the seven sites in the LTGE experiment, establish whether plant growth was density‐dependent (negative slope) (Rees, Condit, Crawley, Pacala, & Tilman, 2001; Umana et al., 2018).

### 2.5 Herbivory and community biomass production

The impact of herbivory on production in the Serengeti was measured during 1974–1976 in the classic study by Sam McNaughton (McNaughton, 1985). Production estimates were made at twenty different sites, many of which are within 2 km of the LTGE experimental sites used to estimate maximum RGR, or *r*. Production was estimated using the moving cage method, which uses temporary, moveable exclosures to estimate biomass production over monthly increments in grazed grassland and permanent exclosures to estimate production in ungrazed control plots (McNaughton et al., 1996). Initial biomass was interpreted as *S_0 _*(1‐*G*) in grazed plots and as S_0_ in permanently fenced plots. Subsequent biomass measurements at time *t* = 20–40 days later were interpreted as *S_g _*(*t*) in temporary fences erected at time *t* = 0, and as *S*(*t*) in the permanent exclosures at time *t*. Production with and without grazing was estimated using Equations 9 and 10 and *ΔP* from Equation 11. Herbivory intensity *G* was measured as 1 – [(biomass in presence of herbivore)/(biomass in permanent exclosures)] at time *t* = 0, per McNaughton (1985). The original biomass and production data were not available, so values were read from graphs in the published paper and are shown in Table 1.

**Table 1:** Data from the McNaughton (1985) 1974–1976 field study used to test predictions of the episodic herbivory model (Figure 6)

Site Code	Description	Mean Annual Rainfall mm	Ungrazed Production g.m^−2^.month^−1^	Ungrazed Peak Biomass g.m^−2^ (S_K_)	Grazed Production g.m^−2^.month^−1^	Initial Biomass/Ungrazed Peak Biomass (S_0_/S_K_)	Grazing Intensity	Δ Production
G1	SouthEast Plains	437	175	902	388	0.81	0.21	213
G2	SouthEast Plains	379	110	690	450	0.84	0.55	340
G3	SouthEast Plains	573	485	745	430	0.35	0.25	−55
G4	Barafu Kopjes	428	195	750	405	0.74	0.62	210
G5	Hill Track	449	405	860	615	0.53	0.38	210
G6	Esoit Ndiakana	655	485	810	550	0.40	0.3	65
G7	Semetu Kopjes	598	390	771	390	0.49	0.18	0
G8	Rongai	719	479	976	480	0.51	0.19	1
G9	Research Center	686	455	951	740	0.52	0.56	285
G10	Kemarishe	727	488	1,006	801	0.51	0.38	313
G11	Musabi Plains	844	580	1,900	950	0.69	0.28	370
G12	Central Hills	632	434	1,231	585	0.65	0.56	151
G13	Togoro Plains	597	288	1,550	290	0.81	0.2	2
G14	Lobo Kopjes	589	280	1,110	330	0.75	0.33	50
G15	Klein's Camp	808	402	1,600	590	0.75	0.4	188
G16	Kuka Hills	793	275	1,350	570	0.80	0.3	295
R1	Gol Kopjes	541	175	742	422	0.76	0.38	247
R2	Kogatende	737	510	645	420	0.21	0.23	−90
R3	Kirawira	987	250	1,210	300	0.79	0.22	50
R4	Masai Mara	1,006	190	1,342	461	0.86	0.58	271

Analysis of patterns in the difference in aboveground monthly production in the presence and absence of herbivory, Δ*P*, was conducted with generalized linear models in SPSS 24. We used a model to explain Δ*P* that included hypothesized independent variables *S*_0_/*S_K_*, *G,* and *G* ^2^ (to capture nonlinear response of Δ*P* to herbivory intensity).

## 3 RESULTS

### 3.1 Thresholds for stimulated production

Solving for the conditions under which Δ*P* > 0 in Equation 7 yields a threshold herbivory intensity, *G’*, below which herbivory stimulates production:

(8) $$G^{\prime }<\left\{\frac{1}{e^{-rt}+\frac{S_{0}}{S_{K}}\left(1-e^{-rt}\right)}+1-\frac{S_{K}}{S_{0}}\right\}$$

**Figure 3:**
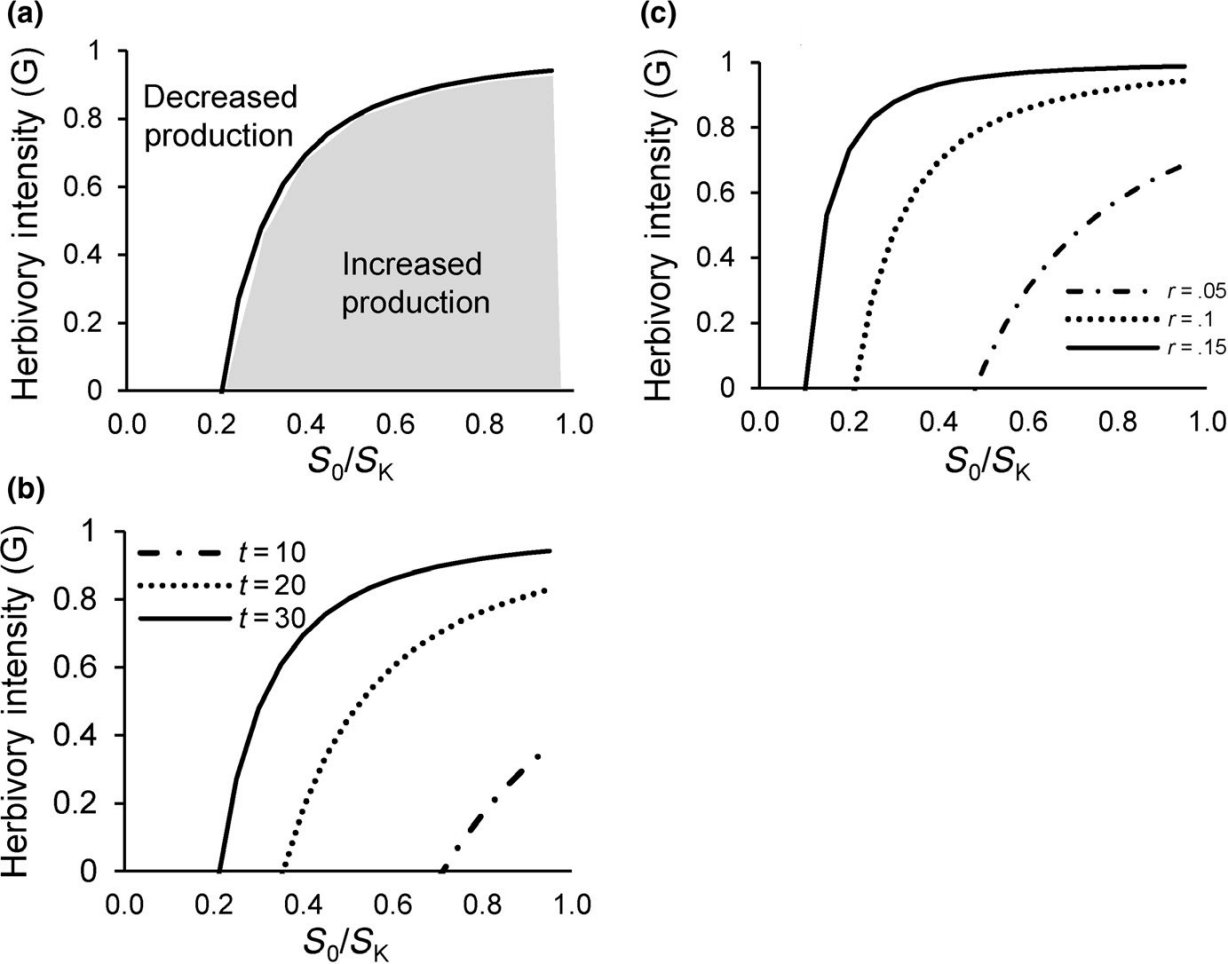
Thresholds of herbivory intensity *G’* below which a grazing event will increase productivity over a time *t*. (a) *G’* in response to increasing initial relative biomass *S*_0_/*S_K_*, where overproduction occurs at all *G* below the curve. Effects on *G’* of (b) increasing time interval *t* following the herbivory, and (c) increasing maximum relative growth ra*te,* or *r*

The relative biomass *S*_0_/*S_K_* is a proportion that reflects how close initial biomass *S*_0_ is to steady‐state biomass in the absence of herbivory *S_K_*. At higher *S*_0_/*S_K_*, the threshold herbivory intensity *G* increases and the magnitude of increase in Δ*P* also increases (Figure 3) because herbivory more strongly weakens density dependence and increases *RGR*. Conversely, herbivory events that occur at low *S*_0_ are more likely to leave remaining biomass at too low a biomass to yield large absolute increases in biomass during growth following herbivory and thus are unlikely to stimulate production.

*G*’ also increases with time following the herbivory event, *t* (Figure 2b), implying that longer rest following herbivory yields a greater chance of overproduction. Defoliated plants that have more time for their higher *RGR* to be in effect effectively “catch up” to the biomass plants would grow to in the absence of herbivory. Conversely, if herbivory events are frequent, with shorter rest times, there is insufficient time for higher *RGR* to produce absolutely more biomass. Herbivory frequency, a largely underappreciated aspect of herbivory, therefore should strongly affect whether overproduction occurs.

**Figure 4:**
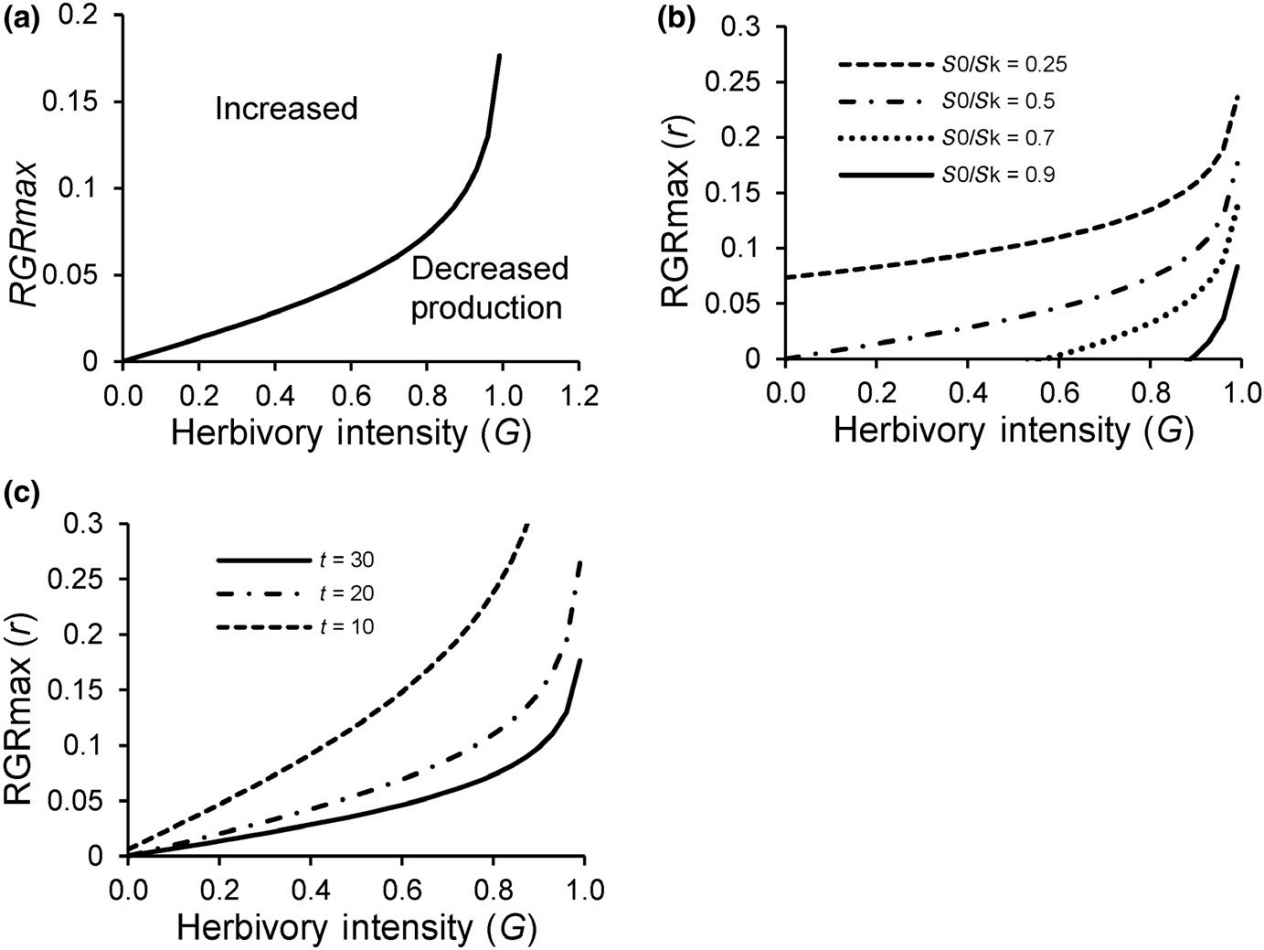
Critical thresholds of *r* for overproduction (Equation 13) as a function of herbivory intensity. (a) Plants with *r* above the threshold will overproduce following herbivory. (b) Biomass relative to that at steady state without herbivory, *S*_0_/*S_K_* and (c) time interval following herbivory, *t*

Maximum *RGR* or *r* strongly influences overproduction. Intuitively, higher *r* should make overproduction more likely because *RGR* is increased more strongly for a given reduction in plant biomass by herbivores if *r* is higher. Figure 3b shows that higher *r* yields a greater range of grazing intensities and initial biomasses under which overproduction can occur, and the magnitude of overproduction also increases with *r* (Figure 2c). Equation 8 can be rearranged to yield a threshold value for *r*, *r’*, as a function of *S*_0_/*S_K_*, *G*, and *t* that must be exceeded for overproduction to occur (Figure 4).

(9) $$r^{\prime }>-\frac{1}{t}\ln\left[\frac{\frac{S_{0}}{S_{K}}\left(1-G\right)}{\left(1-\frac{S_{0}}{S_{K}}\right)\left(G+\frac{S_{K}}{S_{0}}-1\right)}\right]$$

Consistent with the previous predictions, the necessary *r’* to yield overproduction increases at greater herbivory intensity, since a greater reduction in postherbivory biomass *S*_0_(1‐*G*) requires a greater *RGR* to achieve the greater absolute biomass increase necessary for overproduction. Threshold *r’* also declines with increasing initial biomass relative to steady‐state biomass without herbivory (*S*_0_/*S_K_*) (Figure 4b) and increasing time between herbivory events *t* (Figure 4c). Several reviews of plants growing in greenhouses suggest *r* = 0.2–0.4 for a range of annual and perennial herbaceous species. Calculations summarized in Figure 4 suggest that initial *S*_0_/*S_K_* > 0.20 and *t* > 20 would be required for plants to yield overproduction under most herbivory intensities.

### 3.2 Optimized production under herbivory

Figure 2 highlights the phenomenon of “herbivory (grazing) optimization,” that is, an herbivory intensity exists that maximizes production above that expected in the absence of herbivory. Factors that increase the likelihood of overproduction also increase the herbivory intensity that yields maximum production. For longer times (*t* > 20) between herbivory events, optimal herbivory intensity is more than 50% and the increase in production relative to that in the absence of herbivory approaches 50%. This magnitude of difference in production is also obtained when initial biomass approaches 70%–90% of the steady‐state biomass without herbivory *S_K_* (*S*_0_/*S_K_* = 0.7–0.9). Despite the potential for strong overcompensation under such conditions, the model also predicts precipitous drops in productivity with increased herbivory intensities just beyond optimal values (Figures 1c and 2).

### 3.3 Repeated herbivory events

The framework presented thus far allows consideration of the steady state of plant biomass *S_g_*(*t*) Equation 4 under repeated herbivory events of intensity *G* separated by a time interval *t* for plants at a given *r*. This simulates how herbivory occurs under field conditions and explores the more limited possible conditions for overproduction under repeated events. The change in biomass after recovery interval *T* of duration *t* is defined as *S_T+1_*(*t*)−*S_T_*(*t*) and steady state occurs where this difference is zero. Substituting *S*(*t*) for *S*_0_ in Equation 4, one can estimate *S_T+1_*(*t*) and *S*(*t*) for *S_T_*(*t*) under these conditions. Solving for *S*(*t*) yields a relative steady‐state biomass under repeated herbivory S*/*S_K_*.

(10) $$\frac{S^{*}}{S_{K}}=\frac{1}{1-G}\left(1-\frac{G}{1-e^{-rt}}\right)$$

**Figure 5:**
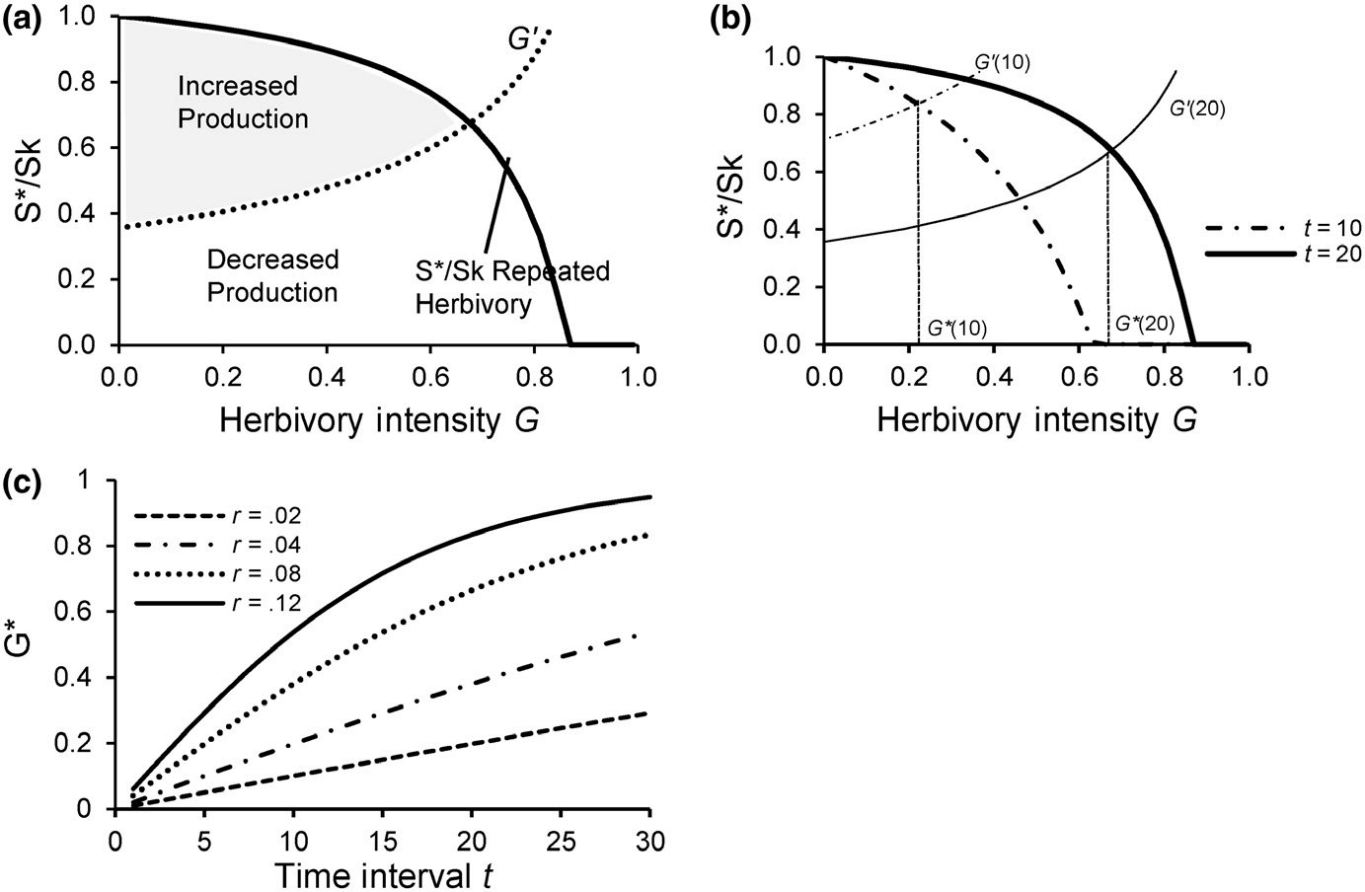
Overproduction under steady‐state conditions of repeated herbivory events separated by time *t*. (a) Relative steady‐state biomass (S*/*S_K_*) under herbivory as a function of herbivory intensity *G* at each event, along with the threshold herbivory for overproduction *G’* (thin dashed curve) for each steady‐state relative biomass. (b) Resulting G* under which overproduction can occur at steady‐state relative biomass under repeated herbivory, determined by the intersection of *G’* (thin curves) (Equation 8) and S*/*S_K_* as a function of *G* (Equation 10) (thick curves) for different time intervals (*t* = 10, alternate dashes; and *t* = 20, solid). (c) Steady‐state threshold herbivory intensity G* as a function of both time interval (*t*) and *r* (Equation 11)

Analysis reveals that above a certain *G*, *G_max_* = 1‐e^−^ *^rt^*, repeated herbivory impacts will reduce biomass to zero, and thus such intensity is not sustainable (Figure 5a). More interestingly, repeated herbivory intensities that can produce sustained overproduction occur under a more restricted set of conditions (Figure 5b). The critical threshold of G* below which overproduction occurs when at steady state during repeated herbivory events is obtained by substituting Equation 6 for *S*_0_/*S_K_* in Equation 8 and solving for *G*

(11) $$G*=\;\left(1-e^{-rt}\right)/\left(1+e^{-rt}\right)$$

Analysis reveals that G* is an increasing function of both *r* and *t*, the time interval between herbivory events (Figure 5c).

### 3.4 Testing with Field Data

**Figure 6:**
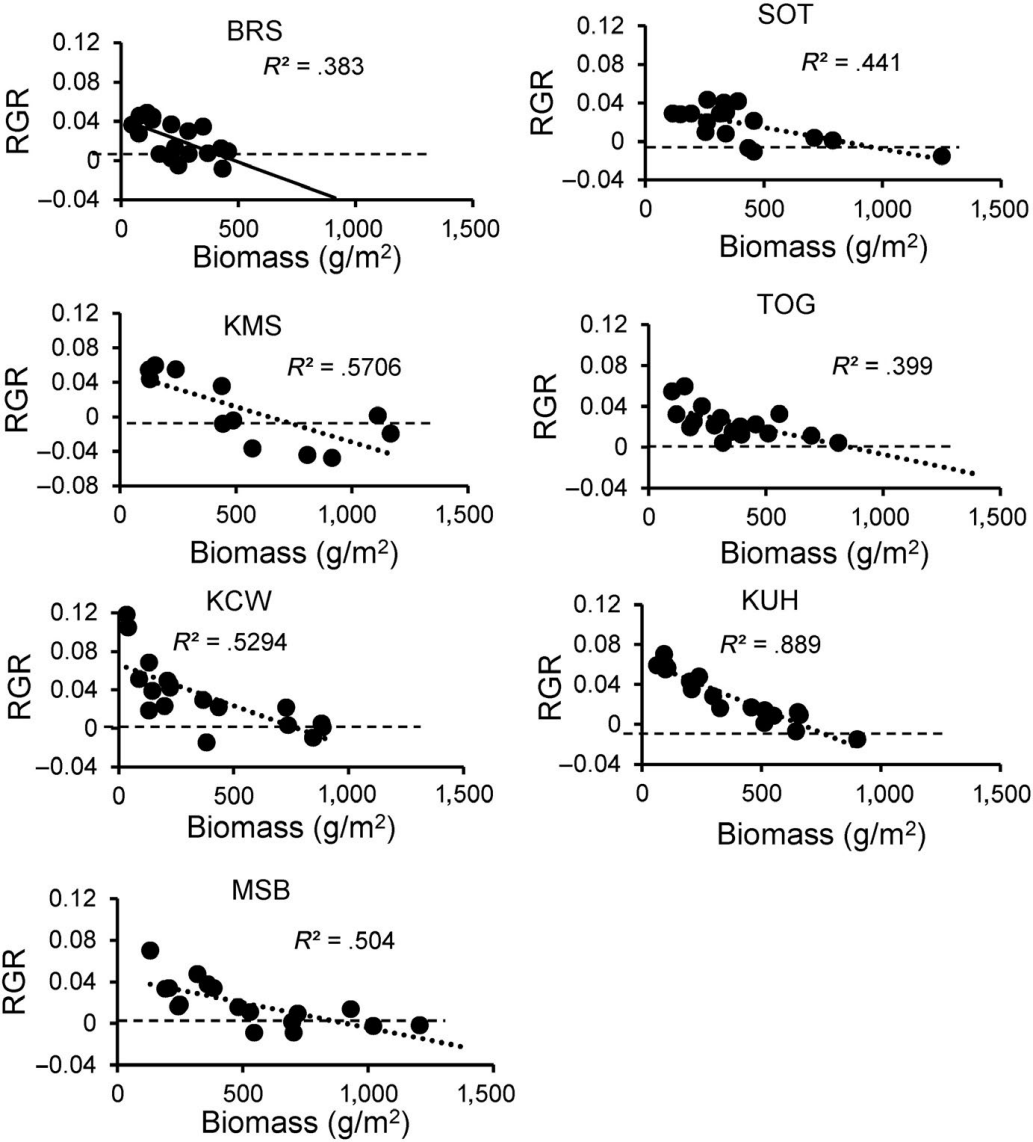
Regressions of community biomass relative growth rate (*RGR*) (*N* = 12) over a month (April‐May or May‐June) versus biomass at the beginning of the month for in three herbivore exclosure plots at each of seven sites in Serengeti National Park in 2000 and 2001

Regressions of *RGR* versus *S_m‐1_* were significantly negative at all seven LTGE sites sampled in 2000–2002 (Figure 6). All intercepts were significantly greater than zero and slopes significantly negative, as expected under moderate to strong density dependence in biomass production (Table 2). Intercepts represent estimates of *r,* and the mean intercept (± SE) across the seven sites was 0.049 ± 0.005 (*N* = 7).

**Table 2:** Regression parameters for community biomass relative growth rate as a function of biomass

Site	Intercept	SE	*P*	Slope × 10^–5^	SE × 10^–5^	*P*	*R* ^2^
BRS	0.042	0.007	<0.0001	−8.8	2.80	0.0061	0.383
KCW	0.066	0.009	<0.0001	−8.5	2.20	0.0006	0.529
KMS	0.053	0.015	<0.0001	−8.2	2.22	0.0006	0.570
KUH	0.063	0.004	<0.0001	−9.4	0.85	<0.0001	0.889
MSB	0.044	0.007	<0.0001	−4.8	1.29	0.0014	0.504
SOT	0.036	0.006	<0.0001	−4.5	1.26	0.0026	0.571
TOG	0.042	0.007	<0.0001	−4.9	1.56	0.0065	0.339

**Figure 7:**
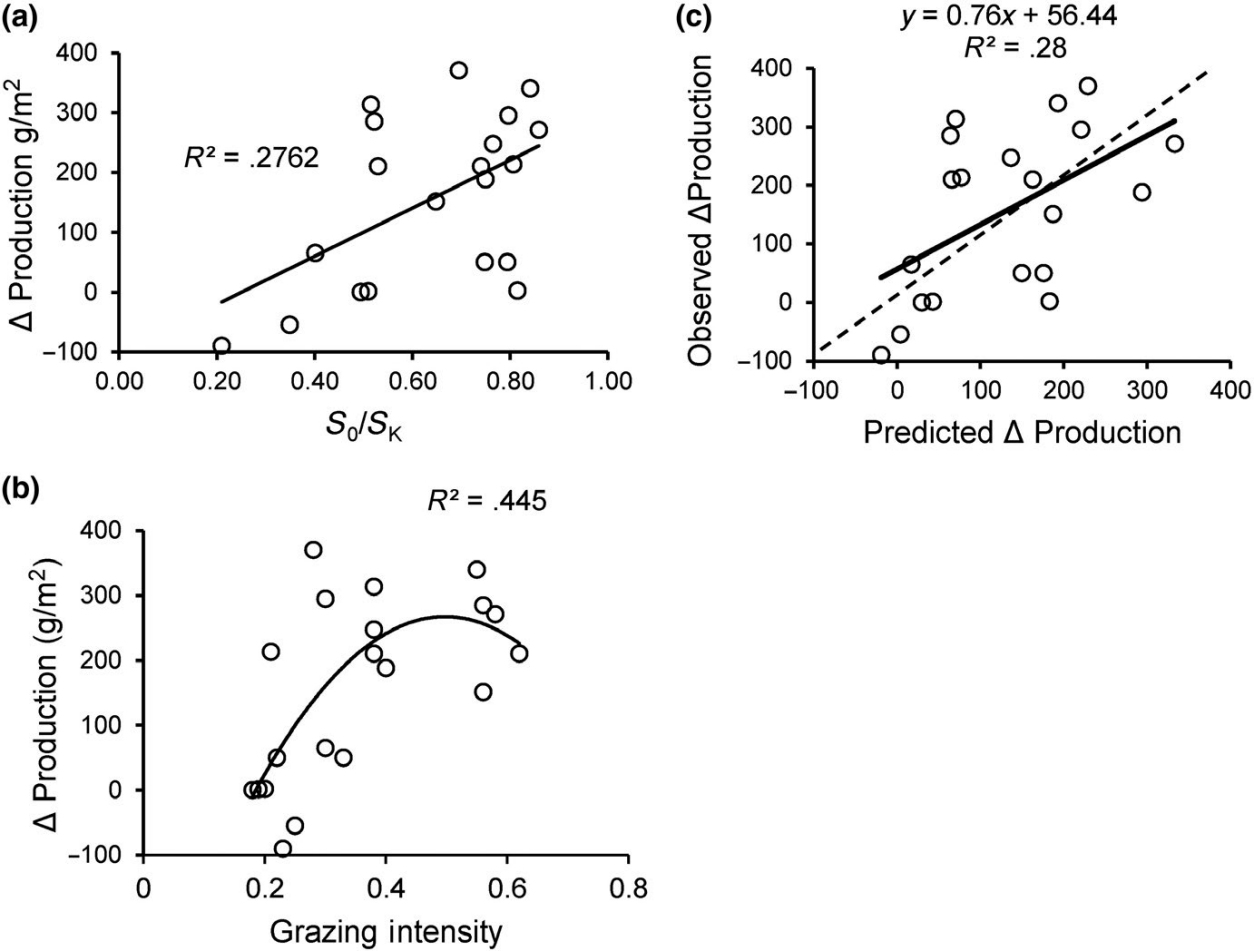
Patterns of herbivory‐induced difference between biomass production in grazed and biomass production in ungrazed (ΔProduction > 0; Equation 11 for *N* = 20 sites in the greater Serengeti ecosystem. (a) ΔProduction exhibits a significant (*p* = .03) association with initial relative biomass *S_0_*/*S_K_*. (b) ΔProduction is associated with measured grazing intensity. Statistical justification of a nonlinear function is provided in Table 3. (c) Association of ΔProduction predicted by the episodic herbivory model, using parameters *r* = 0.06, *t* = 30 days, and initial relative biomass (*S*_0_/*S_K_*) and grazing intensity (*G*) from each of the 20 sites, and observed ΔProductivity for the same sites. The dashed line is the 1:1 relationship

In the McNaughton 1972–1975 field study, Δ*P* (ΔProductivity (Figure 7)) was positive or near zero at 18 of 20 sites. In a generalized linear model, Δ*P* increased significantly with both initial relative biomass (estimated *S*_0_/*S_K_*) (Figure 7a) and a quadratic function of higher herbivory intensity (Table 3; Figure 7b). The model including the quadratic term (with negative coefficient) for herbivory intensity fits the data somewhat better than *S*_0_/*S_K_* plus only a linear term (ΔAICC = 1.271) (Figure 7b), and both the linear term (*p* = .005) and the quadratic term (*p* = .011) were significantly different from zero. The episodic herbivory model (Figures 1c and 2a) predicted both the association of ΔP with *S*_0_/*S_K_* and the nonlinear response of ΔP to herbivory intensity. Predictions of Δ*P* for each site were made using the episodic herbivory model using the average number of days plants were allowed to grow after being caged (*t* = 30), observed *S*_0_/*S_K_* and grazing intensity for each site, and the mean *r* estimate from the y‐intercept of *RGR* versus biomass regressions (Figure 6). The regression of predicted versus observed values was significant (*R* ^2^ = 0.28, *N* = 20, *p* = .01) (Figure 7c).

**Table 3:** Generalized linear models results for the difference in monthly biomass production (ΔProduction) between grazed and ungrazed plots in the Serengeti ecosystem, Tanzania and Kenya, 1972–1975

			Wald Chi‐Square	*df*	*P*
Intercept	−585.379	183.581	10.168	1	0.001
Initial Relative Biomass S_0_/S_K_	306.473	113.993	7.228	1	0.007
Herbivory Intensity, G	2,726.040	975.361	7.811	1	0.005
G^2^	−2,885.859	1,226.149	5.539	1	0.019
Dispersion	7,740.388	2,447.725			

## 4 DISCUSSION

We present here a framework for considering both herbivory impact and time intervals between impacts. This departs from most previous models of plant–herbivore dynamics (Loreau, 2010; de Mazancourt et al., 1998; Ritchie, 2014; Schwinning & Parsons, 1999) where herbivory is assumed to be a continuous process, subtracting biomass on small time scales (d*t*). Continuous herbivory may not capture some more realistic dynamics of plants with their herbivores.

### 4.1 Herbivory and plant production

Our analysis highlights several key predictions that differ from previous evaluations of herbivore effects on plant production. First, short‐term, highly intense herbivory can lead to overproduction as long as herbivory intervals are sufficiently long (Figures 2, 3, 4). Conversely, frequent but less intense herbivory is unlikely to stimulate productivity (Figure 2). Plant traits that contribute to higher *RGR* at low biomass and thus higher maximum *RGR* makes overproduction more likely. Finally, overproduction is more likely when herbivory events occur at plant biomass closer to steady‐state biomass in the absence of herbivory. Consequently, the timing of herbivory during the season may be an important factor in determining whether overproduction occurs.

Alteration by herbivory of plant traits that affect *r* (Adler, Milchunas, Sala, Burke, & Lauenroth, 2005; Diaz et al., 2007; Koricheva & Nykanen, 2004; de Mazancourt et al., 1998), or enhancement of resource availability by herbivores (de Mazancourt et al., 1998; McNaughton et al., 1997; Pastor & Naiman, 1992; Ritchie et al., 1998) are hypothesized mechanisms by which herbivory might stimulate seasonal production. However, a key result of our analysis is that herbivore stimulation of production does not require either of these mechanisms. Indeed *r* in the model is assumed to be constant with or without herbivory, so any increase in production with herbivory does not depend on an effect of herbivory on the inherent capacity for plant growth. Likewise, there is no assumption that plants use stored resources to regrow following herbivory, as biomass accumulation following the herbivory event depends only on growth associated with residual aboveground biomass. Similarly, there is no requirement that herbivory alters carbon allocation in plants, such as might reduce root biomass to compensate for lost aboveground tissue (Milchunas & Lauenroth, 1993). This prediction is supported by multiple studies that find either no or weak impacts of intense aboveground herbivory on root biomass and production (McNaughton, Banyikwa, & McNaughton, 1998; Milchunas & Lauenroth, 1993; Ritchie, 2014).

The episodic herbivory model presented here (Equation 7) suggests that not only is overproduction possible but also it may be expected in situations where herbivory is reasonably intense and initial plant biomass is close to *S_K_*. What is required for significant stimulation of productivity is strong density dependence, or reduction in *RGR* with increasing biomass, herbivory occurring when biomass is near *S*_K_, and long time intervals between herbivory events. This result provides an important alternative prediction to the resource limitation hypothesis (Wise & Abrahamson, 2005, 2008) and may explain why compensation is sometimes observed to be stronger in resource‐poor conditions (Luo et al., 2012; de Mazancourt et al., 1998; Schwinning & Parsons, 1999; Yamauchi & Yamamura, 2004). Recent simulated herbivory studies of both temperate and tropical grass species support these hypotheses (Broadbent et al., 2018, 2019; Mudongo et al., 2016); higher intensity clipping of multiple grass species followed by longer rest periods increased production, while less intense and more frequent defoliation generally either had no effect on or reduced production.

The results of the 1972–1975 Serengeti field study of the effects of migratory grazing mammals on production were consistent with the predictions of the episodic herbivory model. The higher the initial biomass relative to peak or steady‐state biomass without herbivory, the larger the magnitude of overproduction due to herbivory (Figure 6a). Likewise, stimulation of productivity was stronger at intermediate herbivory intensities (Figure 6b). The episodic herbivory model also predicted that overproduction may occur across a wide range of herbivory intensity and initial biomass conditions if *r* > .05 (Figure 4); estimates of daily mean .08 > *r *> .038 across the seven sites sampled in Serengeti during 2000–2002 (Figures 6 and 7) suggest that herbivore stimulation of productivity should be frequent and of relatively large magnitude (Figure 7).

### 4.2 Time intervals between herbivory episodes

Long time intervals between intense herbivory events can occur in a variety of contexts. Herbivores can be highly aggregated in space through a variety of mechanisms. Specialist insects can exhibit a high degree of spatial aggregation (Berenbaum & Isman, 1989; Dennis, Young, & Gordon, 1998; Hassell, Comins, & May, 1991; Hunter & Price, 1998) within a population of hosts, and a given set of hosts can experience high variability in herbivore densities over time (Berryman et al., 1987). Even for small generalist herbivores that show population cycles, such as small mammals (Laine & Henttonen, 1983; Pimm, 1991), intense herbivory can be separated by years of little herbivory. Any of these scenarios can result in long “rest” from herbivory during times when specialist populations are low or concentrated in space on other patches of plants. Likewise herbivores that travel in herds typically present locally high densities that may impose intense herbivory over short periods followed by much longer periods of recovery.

In contrast to these scenarios, frequent, intense herbivory, which may maintain biomass at levels far below *S_K_*, is unlikely to yield overproduction (Figures 2 and 7). Frequent herbivory with short recovery periods maintains plants in a state of high *RGR*. However, insufficient time is available before the next herbivory event for biomass to accumulate at a rate higher than that of plants without herbivory. This is because seasonal production depends on both available biomass and RGR, and under frequent herbivory total plant biomass following herbivory events may be insufficient to yield high biomass accumulation over a short recovery time *t*. Frequent herbivory would be characteristic of systems of high densities of stationary herbivores, such as grazing lawns or shrubs grazed or browsed by resident wild mammalian herbivores (Archibald, Bond, Stock, & Fairbanks, 2005; McNaughton, 1985; Veldhuis, Hulshof, Fokkema, Berg, & Olff, 2016), grazing by diverse fish assemblages in coral reefs (Diaz‐Pulido & McCook, 2003; Hixon, 1996), or continuously grazed livestock systems (Milchunas & Lauenroth, 1993).

Such high‐frequency herbivory events, which yield a repeated or steady‐state biomass prior to subsequent herbivory (Figure 5), yield additional restrictions on herbivory intensity (Figure 6) that depend heavily on time intervals between events. At *t* < 10, overproduction under repeated herbivory across a range of *r* is very unlikely, while at *t* > 20, overproduction can occur under a wide array of herbivory intensities and *r*. However, plant traits or high nutrient availability that yields high *r* can dramatically increase the likelihood of overcompensation under frequent herbivory (Figure 6c). Reviews of plants growing in greenhouses suggest *r* = .2–.4 for a range of annual and perennial herbaceous species (Camargo, Tapia‐Lopez, & Nunez‐Farfan, 2015; Dawson, Fischer, & Kleunen, 2011; Grime & Hunt, 1977). Calculations summarized in Figure 4 suggest that initial *S*_0_/*S_K_* < 0.20 and *t* < 20 would be required for herbivory to decrease production under most grazing intensities.

### 4.3 Implications for conservation and management

The theoretical results presented here may be highly relevant to understanding and managing grazing systems (Briske, 1993; Noy‐Meir, 1993; Zegler et al., 2018). They suggest that systems with migratory grazers with herding behavior, such as occur in the Serengeti (Figure 6), Yellowstone (Frank et al., 2002, 2016), and other natural ecosystems, may be more likely to enhance productivity than systems with “continuous” herbivory by sedentary consumers. Herding increases the short‐term intensity but shortens the length of the herbivory event, and migration may increase the time interval between events. Such conclusions are consistent with studies of mammalian grazing by migratory ungulates in natural ecosystems where migrations are still present (Frank et al., 2002, 2016; de Mazancourt, Loreau, & Abbadie, 1999; McNaughton, 1985; Stewart, Bowyer, Ruess, Dick, & Kie, 2006), but see Knapp et al. (2012).

Experimental studies of “rotational” grazing, which feature variable durations of both herbivory events and rest in fenced paddocks, have yielded mixed results (Briske et al., 2008; Teague, Provenza, Kreuter, Steffens, & Barnes, 2013; Teague et al., 2011). The model outcomes in Figures 2, 3, 4, 5 suggest that many rotational grazing studies may feature grazing “events” that would be better modeled as repeated herbivory events over a portion of the plant‐growing season. Such frequent events might mimic a period of continuous grazing followed by rest. Such a temporal pattern may cause grazing intensity to exceed the threshold G* for overproduction under continuous grazing (small *t*) prior to rest. The mix of continuous grazing and rest may depress biomass too low at the beginning of rest to yield overproduction. Such temporal patterns are avoided by high‐intensity, short duration (few days) grazing events in livestock management systems (Broadbent et al., 2018, 2019; Mudongo et al., 2016; Teague et al., 2013). Previous studies generally lack the necessary data to determine the intensity and time intervals during grazing episodes, so it is not possible to test whether these published rest‐rotation results contradict the predictions of our model. Regardless, the theoretical framework presented here provides a basis for measuring biomass and defining grazing events that could easily be tested in the field (Frank et al., 2016).

Our key result is that herbivory can lead to stimulated plant production when plant growth is resource‐limited and thus density‐dependent, even without herbivore enhancement of plant growth potential (*r*) or nutrient availability. This simple framework of herbivory events punctuated by periods of “rest” for plants that are otherwise resource‐limited can be expanded in future work to consider different herbivory contexts in more detail, such as insect–plant host dynamics, or grazing and browsing by large mammals, or herbivory on woody versus herbaceous plants. Likewise, the model could be modified to evaluate the impact of potential herbivore‐induced modifications of plant traits and plant resources. This episodic herbivory framework provides new hypotheses and the basis for field measurements that could help understand the consequences to both agro‐ecosystems and natural ecosystems of modifications to herbivory, including loss of migration corridors, introduction of exotic herbivores, management of domestic herbivores, and loss of large herbivores.

## CONFLICT OF INTEREST

The authors have no competing interests.

## AUTHOR CONTRIBUTIONS

**Mark E. Ritchie** developed the model, analyzed the data, and wrote the first draft of the manuscript. **Jacob F. Penner** provided conceptual input on the model and manuscript and conducted the literature review.

## AUTHOR CONTRIBUTION

**Mark E Ritchie:** Conceptualization (lead); Data curation (lead); Formal analysis (lead); Funding acquisition (equal); Investigation (equal); Methodology (lead); Project administration (lead); Resources (equal); Software (equal); Supervision (lead); Validation (lead); Visualization (lead); Writing‐original draft (lead); Writing‐review & editing (equal). **Jacob F. Penner:** Conceptualization (supporting); Data curation (supporting); Formal analysis (supporting); Funding acquisition (equal); Investigation (equal); Methodology (supporting); Project administration (supporting); Resources (equal); Software (equal); Supervision (supporting); Validation (supporting); Visualization (supporting); Writing‐original draft (supporting); Writing‐review & editing (equal).

### Open Research Badges

This article has been awarded Open Data and Open Materials Badges. All materials and data are publicly accessible via the Open Science Framework at https://doi.org/10.5061/dryad.qrfj6q5bj.

## Data Availability

All new data not presented in the manuscript have been uploaded to the Dryad database https://doi.org/10.5061/dryad.qrfj6q5bj
